# Amplitude of travelling front as inferred from ^14^C predicts levels of genetic admixture among European early farmers

**DOI:** 10.1038/s41598-017-12318-2

**Published:** 2017-09-20

**Authors:** Fabio Silva, Marc Vander Linden

**Affiliations:** 1 IPHES, Institut Català de Paleoecologia Humana i Evolució Social, Tarragona, Spain and Àrea de Prehistòria, Universitat Rovira i Virgili (URV), Zona Educacional, 4 – Campus Sescelades URV (Edifici W3), 43007 Tarragona, Spain; 2 Faculty of Humanities & Performing Arts, University of Wales Trinity Saint David, Lampeter Campus, Ceredigion, SA48 7ED United Kingdom; 3 UCL Institute of Archaeology, University College London, 31-34 Gordon Square, London, WC1H 0PY United Kingdom

## Abstract

Large radiocarbon datasets have been analysed statistically to identify, on the one hand, the dynamics and tempo of dispersal processes and, on the other, demographic change. This is particularly true for the spread of farming practices in Neolithic Europe. Here we combine the two approaches and apply them to a new, extensive dataset of 14,535 radiocarbon dates for the Mesolithic and Neolithic periods across the Near East and Europe. The results indicate three distinct demographic regimes: one observed in or around the centre of farming innovation and involving a boost in carrying capacity; a second appearing in regions where Mesolithic populations were well established; and a third corresponding to large-scale migrations into previously essentially unoccupied territories, where the travelling front is readily identified. This spatio-temporal patterning linking demographic change with dispersal dynamics, as displayed in the amplitude of the travelling front, correlates and predicts levels of genetic admixture among European early farmers.

## Introduction

The suggested link between a large-scale population movement and the spread of early farming across Europe goes back over more than a century and has since been a constant feature of the corresponding scientific debate^1^. In 1971, while anti-diffusionism was sweeping across archaeological circles, Albert Ammerman and Luigi Cavalli-Sforza showed that a continuous large-scale structured population movement analogous to a wave-of-advance could be inferred from a regression analysis of the then available radiocarbon record^2^. They later suggested that traces of this process of ‘demic diffusion’ could be identified in the spatial pattern of modern genetic variation, with corresponding East-West clines^3^.

Despite having been met by extensive criticism^4,5^, the association between demography and early farming still presents a strong explanatory power to explain the spread of the Neolithic across Europe. The relatively recent possibility of extracting and analysing DNA directly from ancient bone samples has since confirmed that, indeed, the inception of plant and animal domesticates in Europe was paralleled by the arrival of a new population introducing a genetic component so far absent from Mesolithic Europe and ultimately deriving from North-Western Anatolian sources^6,7^. If, at the continental scale, the pattern highlights the proportional contribution of this new genetic component^8^, it is noteworthy that regional studies point to more complicated local demographic scenarios, characterised by varying levels of admixture between this incoming population and local forager communities^9^. Similarly, recent work, coupling the vastly expanded radiocarbon record with new statistical and computational techniques, has equally confirmed the early work by Ammerman and Cavalli-Sforza and also shown higher level of complexity. Firstly, Ammerman and Cavalli-Sforza initially suggested that the spread of early farming was continuous, suggesting an average rate of spread of 1 km/year. Although their 1971 publication already hinted at more variation, the 1 km/year figure has reappeared in analyses using updated radiocarbon datasets^10–13^. Only a few analyses have explored, and observed, that the overall spread of the Neolithic was an uneven process both temporally and spatially, with cycles of expansion and stasis, and local rates of expansion ranging from 0.5 to 2.5 km/year^13–15^. Secondly, the inception of farming has been linked to population booms significant enough to be observed in the bio-anthropological records, leading to what has been called the Neolithic Demographic Transition^16,17^, further underlining the demographic causes of the spread of early farming.

More recently, radiocarbon dates have been used in a novel way. By summing the probability density curves of all known radiocarbon dates for a given area, and controlling for the effects of the calibration curve, one gets an idea of population size changes over time^18–20^. Such Summed Probability Distributions (SPDs for short) have recently been used to infer demographic changes in prehistoric populations in Europe, North and South America^21–24^. Of relevance to the spread of early-farming in Europe was the work of Shennan *et al*. who observed that, shortly after the arrival of farming to several European regions, there seemed to be a population depletion, which they called the ‘boom-bust’ effect^21^.

However, at present, the above results exist but have not been linked. In particular, the demic diffusion and SPD approaches have not been combined, despite the fact that they both rely on the same primary sources: radiocarbon dates. In the present paper, we work under the hypothesis that these analytical tools are sensitive to different facets of the same process of early farming dispersal: diffusion analysis highlights the dynamics and tempo of the travelling wave, whereas the SPDs highlight the connected demographic processes. As they are interrelated, it is important to understand the dispersal dynamics and its tempo so as to aggregate the radiocarbon dates in sensible spatio-temporal windows, from which SPDs are created, therefore ensuring that important patterns are not lost or downplayed by the improper aggregation of the data.

## Methods

**Figure 1:**
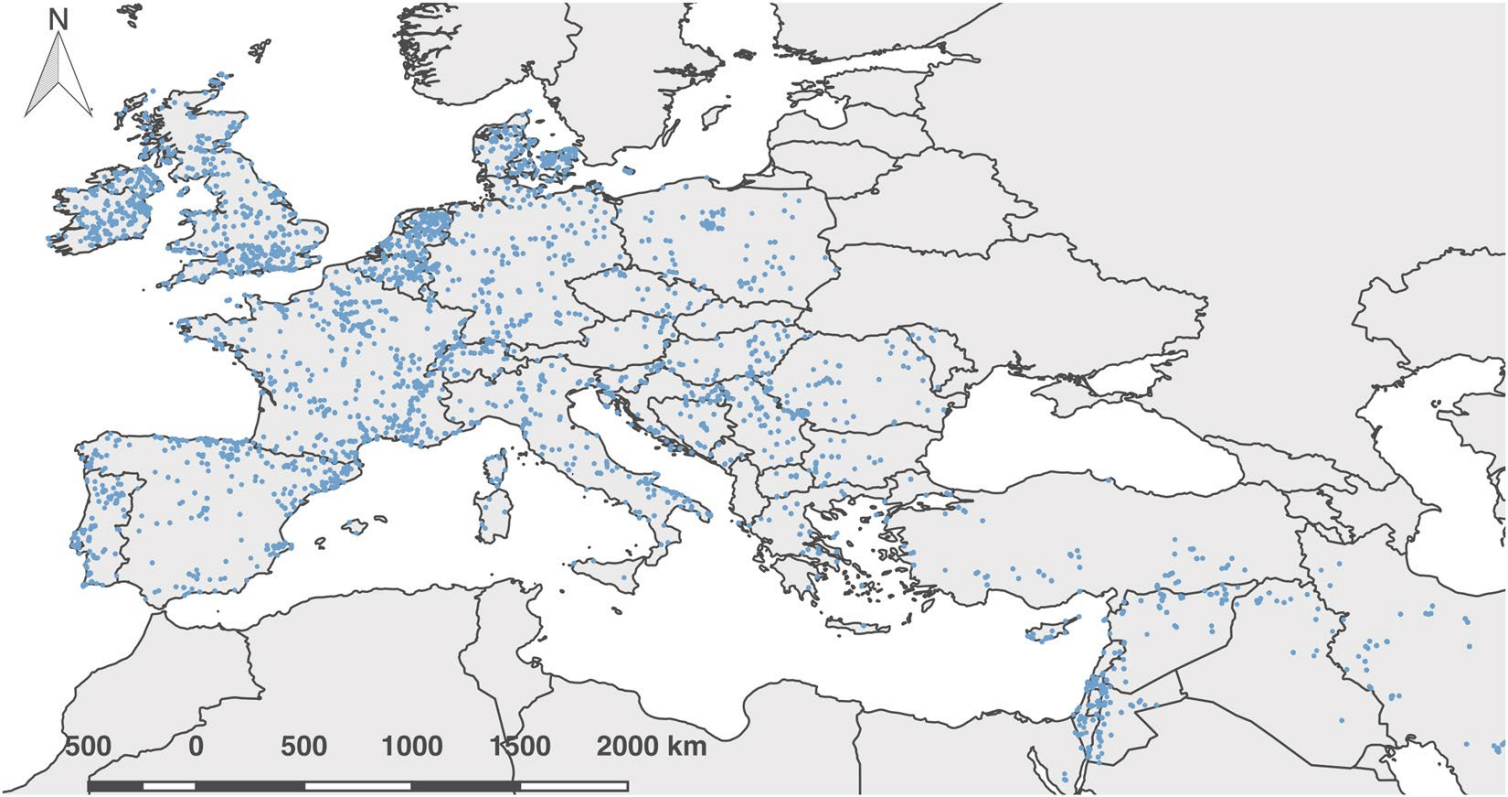
Distribution of dataset used in this research. Blue points indicate archaeological site locations with radiocarbon dates included in the dataset. Map generated with QGIS version 2.14.3-Essen (www.qgis.org).

We assembled a database of 14,535 ^14^C determinations for the Mesolithic and Neolithic periods across the Near East and Europe (Fig. 1). This compilation is based on a series of previous regional surveys of the ^14^C record^25,26^, supplemented by a systematic review of the literature. The upper chronological bracket was set at 12,000 BP, covering the domestication process in the Near East and the majority of the development of Holocene foraging populations in Europe. The lower bracket of 5,000 BP was used for most of the dataset in order to cover two millennia after the local date of introduction of domesticates, enabling the identification of later signals during the Neolithic period. Given the important delay of the introduction of farming in North-Western Europe, a lower bracket of 4,000 BP was used for Ireland, Britain, the Netherlands and Denmark. To achieve a good balance between precision and accuracy, the dataset only includes dates with a standard deviation equal or inferior to 150 radiocarbon years, and all outliers (i.e. dates falling outside the range of existing determinations either at the cultural or site scale) were removed during a phase of critical auditing.

As we are interested in the demographic signals of the advance of early farming we have devised a methodology to track the wave-front and subsequently divide the database into geographical regions based on the dynamics of the wave-front. The dates were calibrated in R^27^ using the Bchron package^28^, and calibration curve Intcal13^29^, and their mode was identified and stored. Subsequently, the Neolithic convex hull, that is the smallest convex polygon that contains all Neolithic data-points within the time range being considered, was calculated for each 100-year interval in the range 12,000–4,000 cal BP. This created a series of stratified polygons corresponding to the increasing surface area covered by the spread of early farming in Europe. A heuristic analysis of the changes in convex hull area through time (see Supplementary Fig. S1) confirms that this spread does not behave like a constantly advancing wave, but rather goes through a finite number of dispersal pulses, interspersed with pauses where occupation of already covered areas is intensified^14,30^.

**Figure 2:**
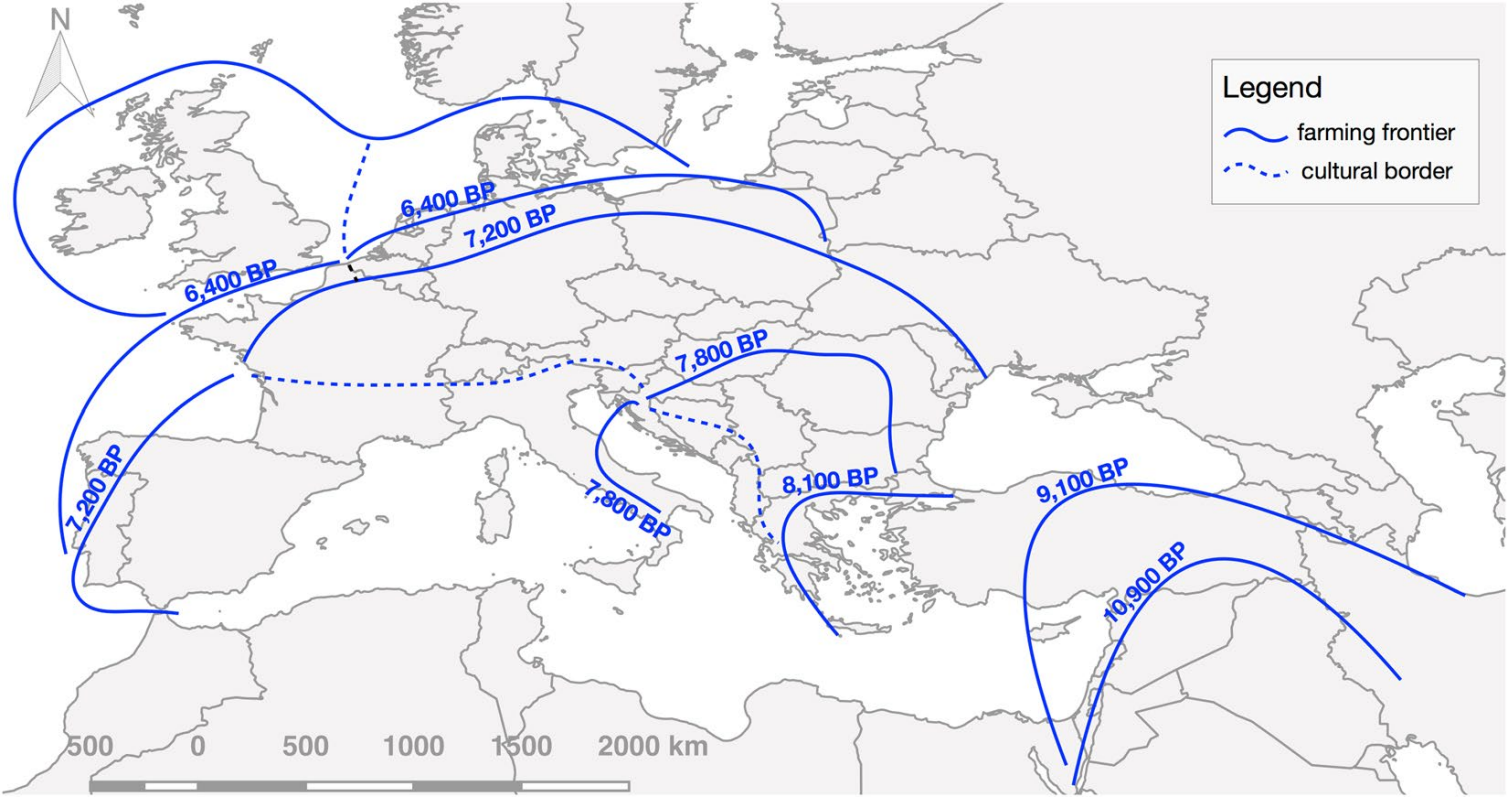
Key pulse-pause episodes in the dispersal of farming in Europe. Solid lines indicate the maximum extent of the spread of farming during each of the pulse-pause episodes identified in this work. Dashed lines correspond to cultural borders. Dates correspond to the time the frontier is first trespassed, initiating a new dispersal pulse in which farming expanded to new territories. Map generated with QGIS version 2.14.3-Essen (www.qgis.org).

We identified six expansion pulses starting at 10,900 cal BP, 9,100 cal BP, 8,100 cal BP, 7,800 cal BP, 7,200 cal BP and 6,400 cal BP, all of which are in agreement with previous estimates^14^. During each of these pulses farming expanded into new territories, represented by six non-overlapping polygons that cover the entire domain (plus a seventh covering the origins of farming in the Levant). The polygons corresponding to the last four pulse episodes were manually split into two regions based on the cultural attributions in the database. This was done to ensure that culturally distinct Neolithization processes and demographic signals were treated separately – e.g. for the LBK of Central Europe and the Cardial of the Western Mediterranean. This resulted in eleven polygons roughly corresponding to the following regions (see Fig. 2): Levant, Anatolia, Aegean, the Adriatic, Balkans, the Western Mediterranean, Central Europe, the Atlantic façade, Northern Europe, United Kingdom and Ireland, and Southern Scandinavia.

This method therefore split the database into regions based on the dynamics of the spread of early farming. To each of these regions the Monte-Carlo Sum Probability Distribution (MCSPD) method was then applied^21^. It involves the calculation of Summed Probability Distributions of radiocarbon dates within a given region, which have been suggested to be proxies for population^21,22^, followed by a statistical test for deviation from a null model of demographic growth, constructed from an exponential fit to the data. We have independently implemented, using R, the MCSPD method as described by Timpson *et al*.^22^, with one modification. Timpson *et al*. included a ‘false positive remover’ as about 5% of false positives will appear to be significant. However, due to the heuristic nature of this remover, and the relative ease with which false positives can be spotted qualitatively, we have opted not to implement this. The output of our algorithm was positively compared to other existing versions of the method^31,32^.

Whereas Shennan and colleagues constructed their null model by fitting to the SPD for the whole of Europe in their time range of interest, we argue that, to identify demographic signals of Neolithization, the null model should be representative of the Mesolithic demographic trend, that is it should be constructed only from Mesolithic data up to the moment of Neolithization. Furthermore, since we are interested in exploring potential regional differences in the dynamics of Neolithization, this trend should be local/regional rather than continental. We have, therefore, fitted an exponential null model to a Mesolithic-only SPD, for each of the convex hull regions of interest.

## Results

**Figure 3:**
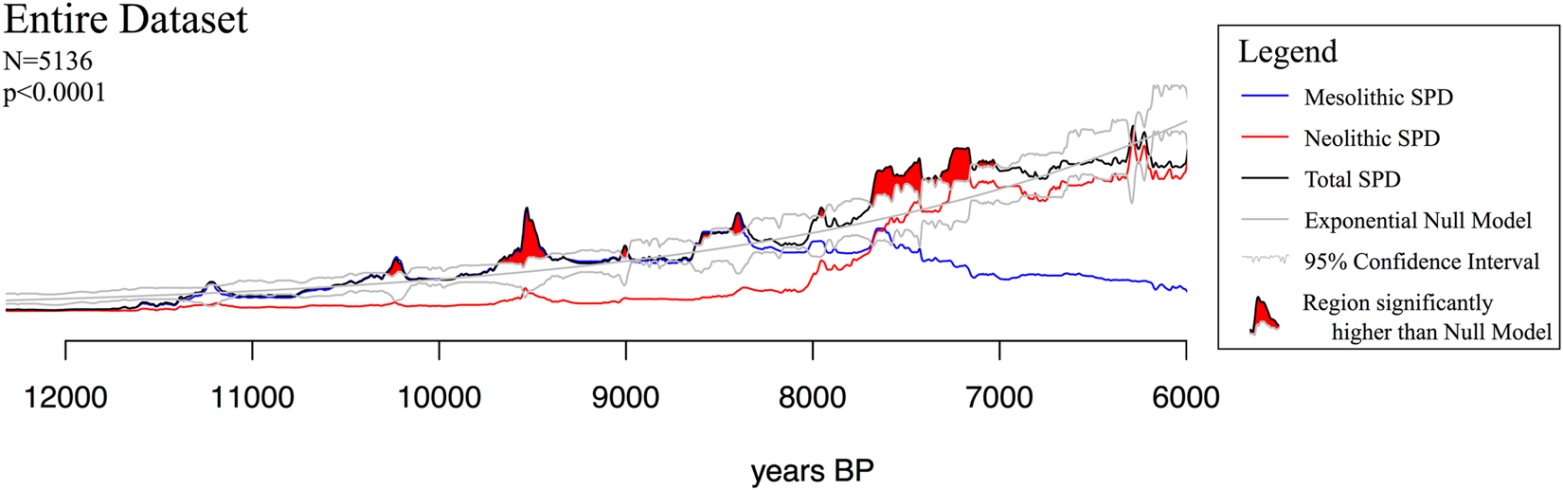
Sum of Radiocarbon Probability Densities (SPD) for the entire dataset (black line). SPDs for the Mesolithic (blue line) and Neolithic (red line) subsets are also shown, as is the fitted null model of exponential growth (grey line) and its 95% confidence interval. Values above this confidence interval are highlighted in red. Figure generated with R version 3.3.2 (www.r-project.org).

**Figure 4:**
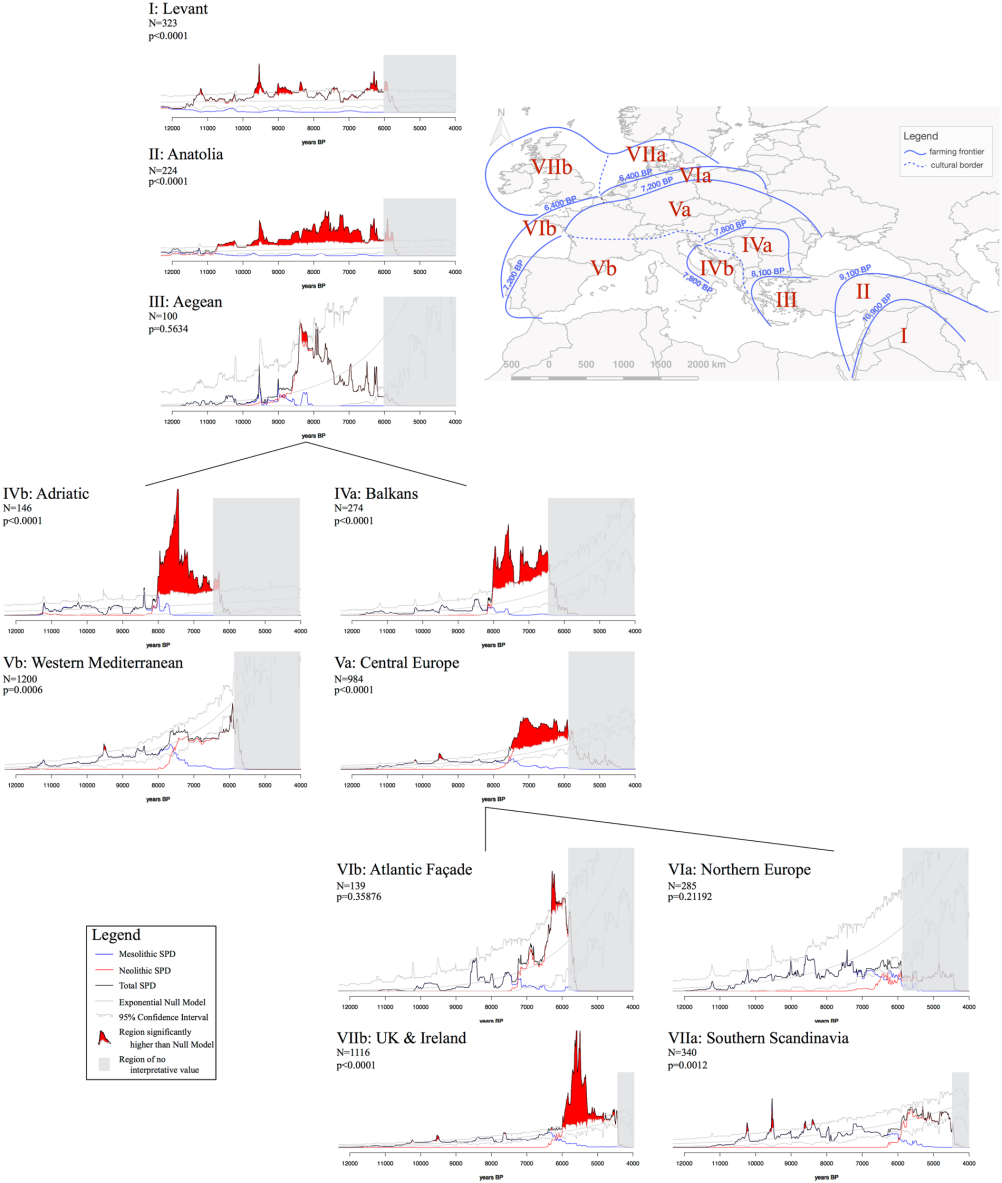
Sum of Radiocarbon Probability Densities (SPD) for each region. The regions are identified in the map at the top-right. SPDs for the local Mesolithic (blue line) and Neolithic (red line) subsets are shown, as is the fitted null model of exponential growth (grey line) and its 95% confidence interval. Values above this confidence interval are highlighted in red. Sub-sampled time periods, corresponding to the end of the sampling range, hold no interpretative value and are, therefore, covered in grey shading. Map generated with QGIS version 2.14.3-Essen (www.qgis.org) and other figures generated with R version 3.3.2 (www.r-project.org).

The results are shown in Figs 3 and 4, where all the SPDs were appropriately rescaled so as to be comparable across the different regions. The reported *N* values are number of site-phases identified by the MCSPD ‘binner’ function, whereas the p-values correspond to Timpson *et al*.’s ‘global p-value’. Figure 3 shows the global SPD for Europe and the Near East. It displays an essentially continuous exponential growth with no long-term breaks from the Mesolithic trend. However, there are a few regions significantly above the null model, some of which might be false positives. For example, the large peak around 9.5 k cal BP is directly correlated with a peak in the calibration curve, and therefore likely to be a false positive. On the other hand, the 500-year region that starts at about 7.8 k cal BP covers the bulk of the farming spread through Europe, and therefore cannot be so easily ignored. But despite this potentially significant fluctuation, the overall trend for the European meta-population is that of a steady exponential growth at an annual rate of 0.0438 ± 0.0002, or 0.0339 ± 0.0002 when corrected for taphonomic bias^23,33^ (see Supplementary Table S1 and Supplementary Fig. S2 for growth rate values). To evaluate the importance of these fluctuations, as well as others that might remain unseen when looking at such large scales, it is important to break this down geographically by following the link between demography and spread of farming, as explained in the *Materials and Methods* section.

The regional SPDs (Fig. 4) show clearly differentiable patterns that can be divided into three regimes. Firstly, for the Near East, Neolithization involves the appearance of statistically significant deviations from the Epi-Palaeolithic trend. Rather than being sharp changes, these are better explained by a change from quasi-stationary trends (annual growth rates of 0.0035% and 0.0109%, uncorrected), suggestive of populations at or near carrying capacity, to a steady increase (especially in Anatolia, SPD II), suggesting a boost in carrying capacity which would be expected from the innovation of farming and animal husbandry^34,35^.

A second regime is displayed in regions with a low Mesolithic population, where the introduction of farming is characterised by statistically significant ‘booms’. This is seen firstly in the Balkans (IVa) and Adriatic (IVb) regions, followed by Central Europe (Va) and United Kingdom and Ireland (VIIb). When these SPDs are staggered, a spatio-temporal sequential pattern of ‘booms’ emerges, where a boom in a region is preceded by a drop in the preceding region (see Supplementary Fig. S3). These could, at first glance, be interpreted as ‘busts’, but their spatio-temporal sequential pattern suggests that such trends in the radiocarbon record should not be interpreted independently of the spatial processes that generated them, in this case the dynamics of the expansion of early farming, a point which we will return to in the discussion.

Regions that fall under the third regime, on the other hand, have a larger Mesolithic population and the introduction of farming does not display a statistically significant deviation from the local Mesolithic growth trend. These include the Western Mediterranean (Vb), Northern Europe (VIa) and Southern Scandinavia (VIIa), as well as, possibly, the Aegean (III) and Atlantic (VIb) regions. The last two do display short ‘booms’ above the null model’s confidence interval, however, due to their small sample sizes (100 and 139 respectively) and high p-values (0.5634 and 0.3588 respectively), these are likely to be false positives and, therefore, we will avoid over-interpreting them. Two of these regions yield p-values that could otherwise be considered significant (0.0006 and 0.0012 for regions Vb and VIIa respectively). However, these values are still much larger than those found in the regions belonging to the second regime and a cursive look at the corresponding SPDs shows that the largest deviations from the exponential growth model correspond to a sharp peak around 8,200 cal BP, a well-known climatic event that might have had demographic consequences in these regions of high density of hunter-gatherers, and therefore does not correspond to the introduction of farming^36^.

## Discussion

In line with previous work^23^, we do not find evidence for any long-term major demographic upswing related to the introduction of farming across the research area during the period between 12,000 and 6,000 cal BP. The overall trend is rather one of continuous exponential growth since the early Holocene, with some short-to-medium term fluctuations, just as observed for North America^23^. This being said, the results also demonstrate a clear, punctuated link between demographic changes and farming, happening during the transition period to the new productive economy. This Neolithic Demographic Transition, already observed in palaeoanthropological data^16,34^, presents some regional patterning observed both in terms of the tempo of the expansion of farming and corresponding fluctuations of the SPDs.

The SPDs for the Levant and eastern Anatolia suggest that the domestication process per se was not mirrored by any sudden increase of the population, but rather a constant rise stretched over a long period of time^35,37^. Recent aDNA research indicates the presence of several populations with distinct genetic signatures, centered upon Anatolia/Levant, and Iran/Caucasus^8^. Each of these groups presents continuity during the transition from foraging to farming, and admixture only happened to significant levels later during the Copper and Bronze Age^38,39^. Whilst the SPDs for western Anatolia and the Aegean basin are difficult to interpret given the small sample size, it is clear the diffusion of farming across this area happens relatively quickly during the first half of the 9^th^ millenium cal BP^40–42^, and upon a background of limited preceding Mesolithic population^43^. This process exhibits regional variation, for instance in terms of stock-breeding strategies^44^, but from a population point of view, the picture looks different. Available aDNA sequencing from a cluster of western Anatolian sites indicates a coherent genetic identity, showing links with the Neolithic Levant populations^8^. It is this population that is responsible for the introduction of farming into Greece and, beyond, to the rest of Europe^7,8,39,45^.

From the southern Balkans onwards, early farming spread along two main corridors across Europe: one along the Danube-Rhine axis, and another across the Mediterranean. Both streams are associated with distinct archaeological assemblages and agricultural practices^46^. Our analysis shows that this divergence also concerns demography. The SPDs for the Balkans present a dramatic rise between 8,000 cal BP and 7,600 cal BP, indicating an incoming population – the travelling front – that replaces local foragers, only identified in specialised ecological niches such as the Danube Iron Gates. This interpretation is confirmed by aDNA studies which show very limited level of admixture with local foragers^47,48^. A single Eneolithic sample from Romania however suggests an increase in the proportion of Mesolithic genetic component, suggesting the existence of a complex population landscape in the area^49^. Around 7,600–7,500 cal BP, the SPDs for the Balkans present a marked dip, possibly affected by limited radiocarbon sampling^50^. Keeping this potential limit in mind, it is however noteworthy that this apparent ‘bust’ coincides with the start of the expansion of farming across central Europe. We consider that this correspondence points only to a limited depletion of the population in the southern Balkans, linked to the the movement of the travelling front of the wave-of-advance, rather than to a collapse related to changes in farming productivity. The amplitude of this expansion episode, associated with the LBK culture, is exceptional as it covers an area stretching from western Ukraine to the Paris basin and corresponds to a steep rise in the SPDs, which contrasts with the limited signal associated with the local Mesolithic. As for the Balkans, this demographic boom corresponds to the arrival of a large population, characterised by the same North-Western Anatolian genetic component with very limited level of admixture with the last foragers^6^.

The second stream of farming diffusion starts in the Adriatic basin. The SPDs point to a demographic peak by 8,000 cal BP associated with the onset of farming practice, although the extent of this expansion is relative given the small corresponding area. The current absence of aDNA makes any interpretation in population terms difficult^51^, but the SPDs indicate that the Neolithic population was larger than the Mesolithic one, sparsely identified in the Eastern Adriatic during the centuries preceding the introduction of farming^52^, and mostly confined to the Alpine region in the western Adriatic^53,54^. The demographic rise comes to an abrupt halt by 7600–7500 cal BP, at the same time as farming expands across the western Mediterranean. As for inland Europe, we suggest that this chronological coincidence points to the travelling front of the wave-of-advance. In this case however, the SPDs indicate that the front was limited in size and met a well-established foraging population, although regional variations in this process are likely^55^. This variation is reflected in the aDNA record, which unambigously shows the introduction of a new population, as in Sardinia for instance^56,57^, and which presents in Iberia a high level of admixture between the new North-Western Anatolian and the Western European Mesolithic genetic components^58^.

The spread of farming across Europe comes to a halt towards the beginning of the 7^th^ millennium cal BP and only resumes in southern Scandinavia, Britain and Ireland nearly a millennium later. This renewed expansion is played on two modes, each with distinct spatial, demographic and genetic signatures. Late foragers in both northern Europe and southern Scandinavia are well-recorded, and known to have been in regular contact with farming groups further south^59^. For both areas, the SPDs do not show any significant rise in population related to the shift to agriculture, but rather a continuous growth within the expected rate for the local Mesolithic population. This is in agreement with the suggestion that domesticated plants and animals were assimilated by local foraging groups, with only small-scale inward migration of pioneering farmers from central Europe^60^, as also reflected by the changing level of admixture between Mesolithic and Neolithic genetic components^61^. Noticeably, this gene flow was not unidirectional towards the North, but also in a reverse direction with the contemporaneous reintroduction in central Europe of haplotypes associated with Mesolithic groups^62–64^. Britain and Ireland exhibit a different situation where, upon a background of low, but not non-existent^65^, Mesolithic population, the introduction of farming during the early centuries of the 6^th^ millenium cal BP coincides with a steep demographic ‘boom’ seen in the SPDs. This event has been identified before and interpreted as the sign of a large-scale migration^19^. Corresponding aDNA evidence is still rare for Britain and Ireland, but analysis of a single Middle Neolithic Irish individual seems to confirm the large size of this incoming population^9^, which introduced in Ireland the same genetic component of Anatolian origin as in continental Europe. However, this sample also presents a large admixture with Western European Mesolithic genetic components. In the absence of further data, it is impossible to discern whether this admixture corresponds to local interactions between the indigenous foragers and Neolithic migrants, or was already a feature of the incoming population, since comparable admixture has been recorded for the mid 7^th^ millennium cal BP in the Paris basin^66^, a likely area of origin for this migrant population^67^. This major episode of growth is followed by a ‘bust’, sometimes interpreted as a depletion of Irish and British Neolithic populations^21^. This hypothesis, and especially the magnitude of the suggested demographic process, is however challenged, as many other factors are likely to have cumulatively affected the shape of the SPDs, including downfall of agricultural systems^68^, environmental factors^69^ and the changing nature of the archaeological record^70^.

## Conclusion

Our analysis of the radiocarbon record between 12,000 and 4,000 cal BP indicates that the spread of early farming was not a continuous process, but was rather structured by a series of punctuated expansions interspersed with periods of stasis of changing duration. In demographic terms, we identified three regimes corresponding, respectively, to: (i) the centre(s) of farming innovation, (ii) its expansion to essentially unoccupied territories, and (iii) its expansion to territories with high densities of hunter-gatherer populations.

The previously identified ‘boom-bust’ demographic pattern is only observed for the second regime, roughly corresponding to the continental stream of Neolithisation, as well as the United Kingdom and Ireland, where the evidence is strongest. However, contrary to its interpretation as episodes of population growth and sudden collapse, their sequential staggering through time and space, identified here for the first time, suggests that they rather correspond to the demographic signature of a travelling wave-front. In this interpretation, the ‘boom’ is linked to the arrival of new people, whilst the ‘bust’ must be understood as due to outgoing migrants, resuming their spread into a new region. This interpretation is consistent with the expected properties of demic diffusion, as only the wave-front experiences a noticeable demographic pressure, whilst the meta-population follows a neutral growth curve^3^, as indicated by the SPD across the entire research area.

These radiocarbon-based interpretations correlate well with the available aDNA evidence, which identifies an overwhelming genetic input from Near Eastern farmers in regions corresponding to our second regime. On the other hand, in the Western Mediterranean and Southern Scandinavia, which provide the strongest third regime signals, we observe a continuous growth that follows the trend set by the well-documented local Mesolithic populations. In these regions, a new population is still documented by aDNA but their relative size is not as significant. We therefore consider that our method offers a robust framework to test archaeological hypotheses regarding cases of demic diffusion for which aDNA evidence is limited or not yet available^71^. Lastly, it must be stressed that the presence, or absence, of a demic wave-of-advance does not provide an explanation for the material variability associated with the spread of early farming across Europe. Likewise, more work is needed to characterise the factors affecting the changing tempo of this diffusion.

## Electronic supplementary material

Supplementary Information
