# Author Correction: Amplitude of travelling front as inferred from ^14^C predicts levels of genetic admixture among European early farmers

**DOI:** 10.1038/s41598-018-30976-8

**Published:** 2018-08-21

**Authors:** Fabio Silva, Marc Vander Linden

**Affiliations:** 1 IPHES, Institut Català de Paleoecologia Humana i Evolució Social, Tarragona, Spain and Àrea de Prehistòria, Universitat Rovira i Virgili (URV), Zona Educacional, 4 – Campus Sescelades URV (Edifici W3), 43007 Tarragona, Spain; 2 Faculty of Humanities & Performing Arts, University of Wales Trinity Saint David, Lampeter Campus, Ceredigion, SA48 7ED United Kingdom; 3 UCL Institute of Archaeology, University College London, 31-34 Gordon Square, London, WC1H 0PY United Kingdom

Correction to: *Scientific Reports* 10.1038/s41598-017-12318-2, published online 20 September 2017

In this Article we uncritically employed the terms ‘travelling wave’ and ‘travelling wave-front’ to describe the spread of the Neolithic. There is, however, a distinction between these terms which relate to two different models of dispersal. In Archaeology, the term ‘wave-front’ has been largely associated with the demic diffusion model of Ammerman and Cavalli-Sorza (1971) where the population of previously occupied territories is kept at carrying capacity even after the wave-front passes through them. By contrast, in a travelling wave model the population of previously occupied territories would decrease back to neutral growth levels once the wave moves out of those territories.

As a result, in the Conclusion section,

“However, contrary to its interpretation as episodes of population growth and sudden collapse, their sequential staggering through time and space, identified here for the first time, suggests that they rather correspond to the demographic signature of a travelling wave-front. In this interpretation, the ‘boom’ is linked to the arrival of new people, whilst the ‘bust’ must be understood as due to outgoing migrants, resuming their spread into a new region. This interpretation is consistent with the expected properties of demic diffusion, as only the wave-front experiences a noticeable demographic pressure, whilst the meta-population follows a neutral growth curve^3^, as indicated by the SPD across the entire research area.”

should read:

“However, contrary to its interpretation as episodes of population growth and sudden collapse, their sequential staggering through time and space, identified here for the first time, suggests that they rather correspond to the demographic signature of a travelling wave. In this interpretation, the ‘boom’ is linked to the arrival of new people, whilst the ‘bust’ must be understood as due to outgoing migrants, resuming their spread into a new region. This interpretation is not consistent with the expected properties of demic diffusion, as only the regions being passed by the travelling wave experience a noticeable demographic pressure, whilst the meta-population follows a neutral growth curve^3^, as indicated by the SPD across the entire research area.”

The conclusions of the Article are unaffected by this change. The authors apologise for the error and any confusion caused.

