# Supplementary material for: Amplitude of travelling front as inferred from 14C predicts levels of genetic admixture among European early farmers
Source: Sci Rep. 2017 Sep 20;7:11985. doi: 10.1038/s41598-017-12318-2 (PMC5607300; doi:10.1038/s41598-017-12318-2)
Supplement: Supplementary file 1 — Supplementary Information [file 41598_2017_12318_MOESM1_ESM.pdf]

Supplementary Information for
Amplitude of travelling front as inferred from 14C predicts levels of genetic admixture among European early farmers

Fabio Silva and Marc Vander Linden

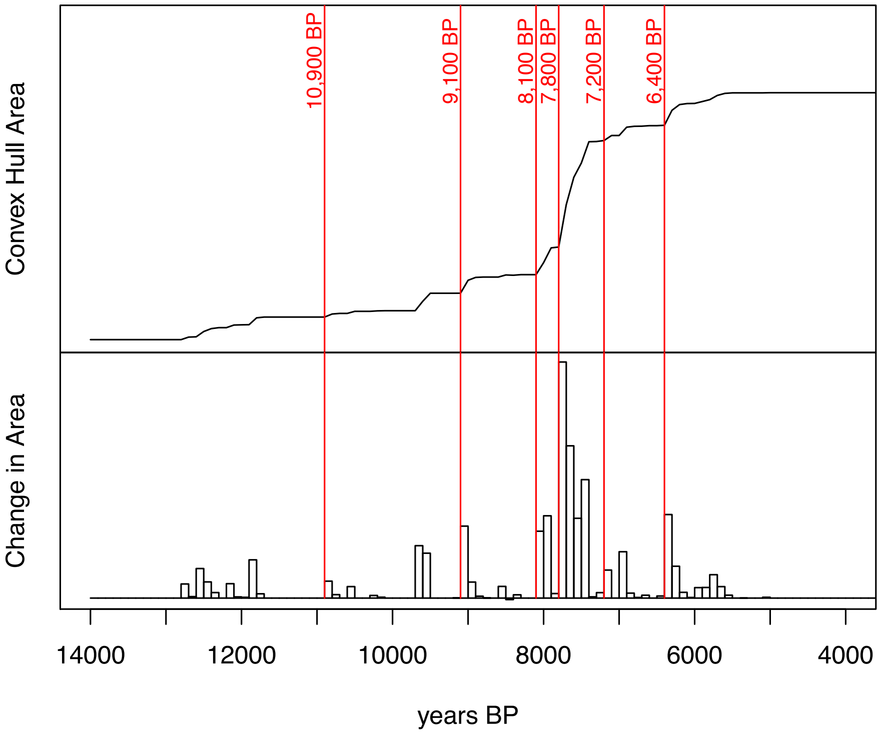

Supplementary Figure S1 – Heuristic analysis of the changes in Neolithic diffusion area through time, showing the area of the convex hull (top) and the change in area (bottom). The latter shows distinct periods of expansion interspersed with pauses. The vertical lines show the onset of the major expansion episodes into new territories.

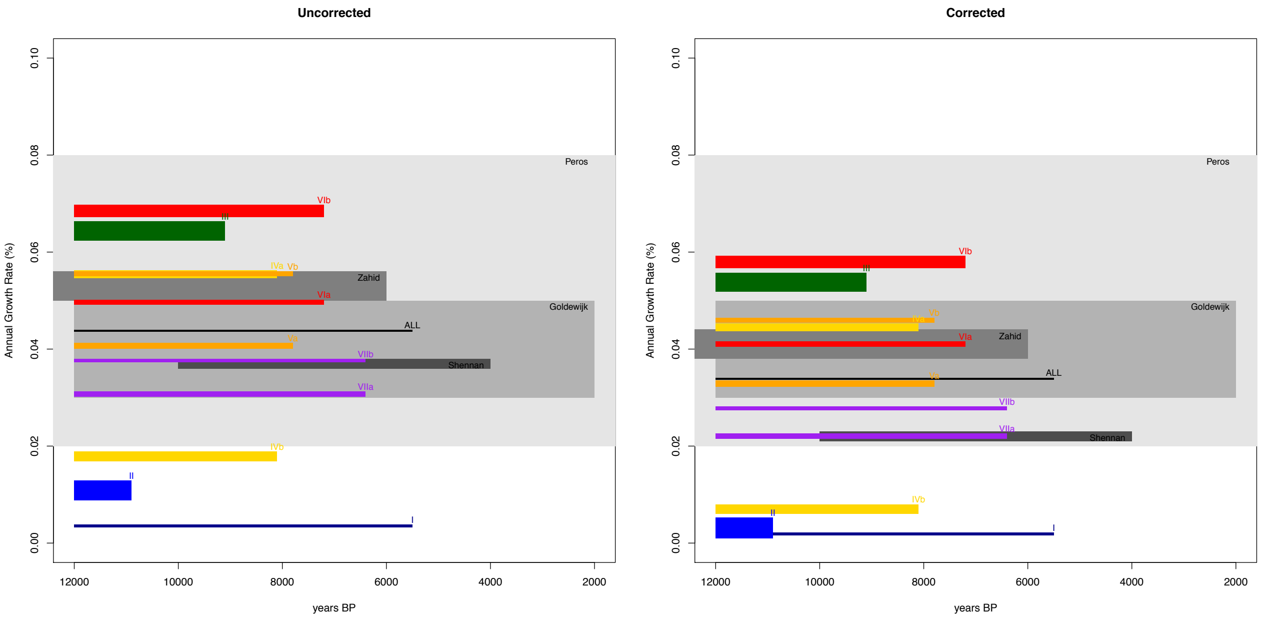

Supplementary Figure S2 – Observed growth rates (coloured bars) of the null models which were fitted to the local Mesolithic SPDs both uncorrected (left) and corrected for taphonomic bias (right), compared to other values found in the literature (grey bars). The width of each bar represents one standard deviation, and each bar extends over the period in which the null model was fitted.

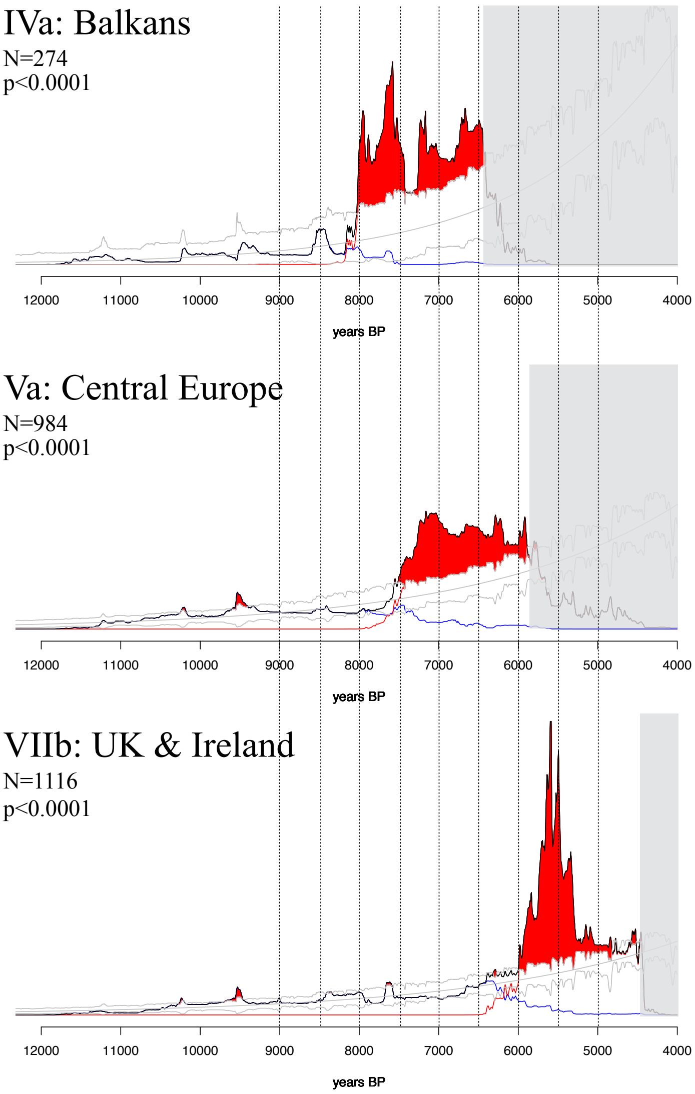

Supplementary Figure S3 – Sum of Radiocarbon Probability Densities (SPD) for the Balkans, Central Europe and United Kingdom & Ireland regions, with overlaid 500-year intervals (vertical dotted lines) to emphasize their spatio-temporal staggering. For example the dip around 7,500 cal BP in the Balkans matches the expansion of farming into Central Europe. The latter slowly falls down to null model values by about 6,000 cal BP, which matches the expansion into the United Kingdom & Ireland.

Database	Number of 14C dates	MCSPD p-value	Growth Rate	Growth Rate corrected for taphonomic bias
I - Levant	1,530	<0.0001	0.00354 ± 0.00028	0.00186 ± 0.00026
II - Anatolia	1,098	<0.0001	0.01088 ± 0.00206	0.00312 ± 0.00212
III - Aegean	567	0.5634	0.06431 ± 0.00200	0.05375 ± 0.00195
IVa - Balkans	1,219	<0.0001	0.05546 ± 0.00080	0.04451 ± 0.00079
IVb - Adriatic	315	<0.0001	0.01790 ± 0.00098	0.00702 ± 0.00097
Va - Central Europe	2,359	<0.0001	0.04066 ± 0.00059	0.03288 ± 0.00062
Vb - Western Med	2,289	0.0006	0.05549 ± 0.00049	0.04599 ± 0.00051
VIa - Northern Europe	910	0.21192	0.04969 ± 0.00051	0.04109 ± 0.00052
VIb - Atlantic Façade	243	0.35876	0.06849 ± 0.00126	0.05795 ± 0.00124
VIIa - Southern Scandinavia	757	0.0012	0.03076 ± 0.00052	0.02206 ± 0.00055
VIIb - UK & Ireland	3,244	<0.0001	0.03761 ± 0.00035	0.02780 ± 0.00037
Entire Dataset	14,535	<0.0001	0.04381 ± 0.00019	0.03392 ± 0.00020

Supplementary Table S1 – Table showing the number of 14C dates used, obtained MCSPD p-value and growth rates, including their uncertainty, both uncorrected and corrected for taphonomic bias (see main text), for each region and for the entire dataset.
